# Supplementary material for: From Double-Strand Break Recognition to Cell-Cycle Checkpoint Activation: High Content and Resolution Image Cytometry Unmasks 53BP1 Multiple Roles in DNA Damage Response and p53 Action
Source: Int J Mol Sci. 2022 Sep 5;23(17):10193. doi: 10.3390/ijms231710193 (PMC9456172; doi:10.3390/ijms231710193)
Supplement: Supplementary file 1 [file ijms-23-10193-s001.zip › SupplementaryFigureS1.pdf]

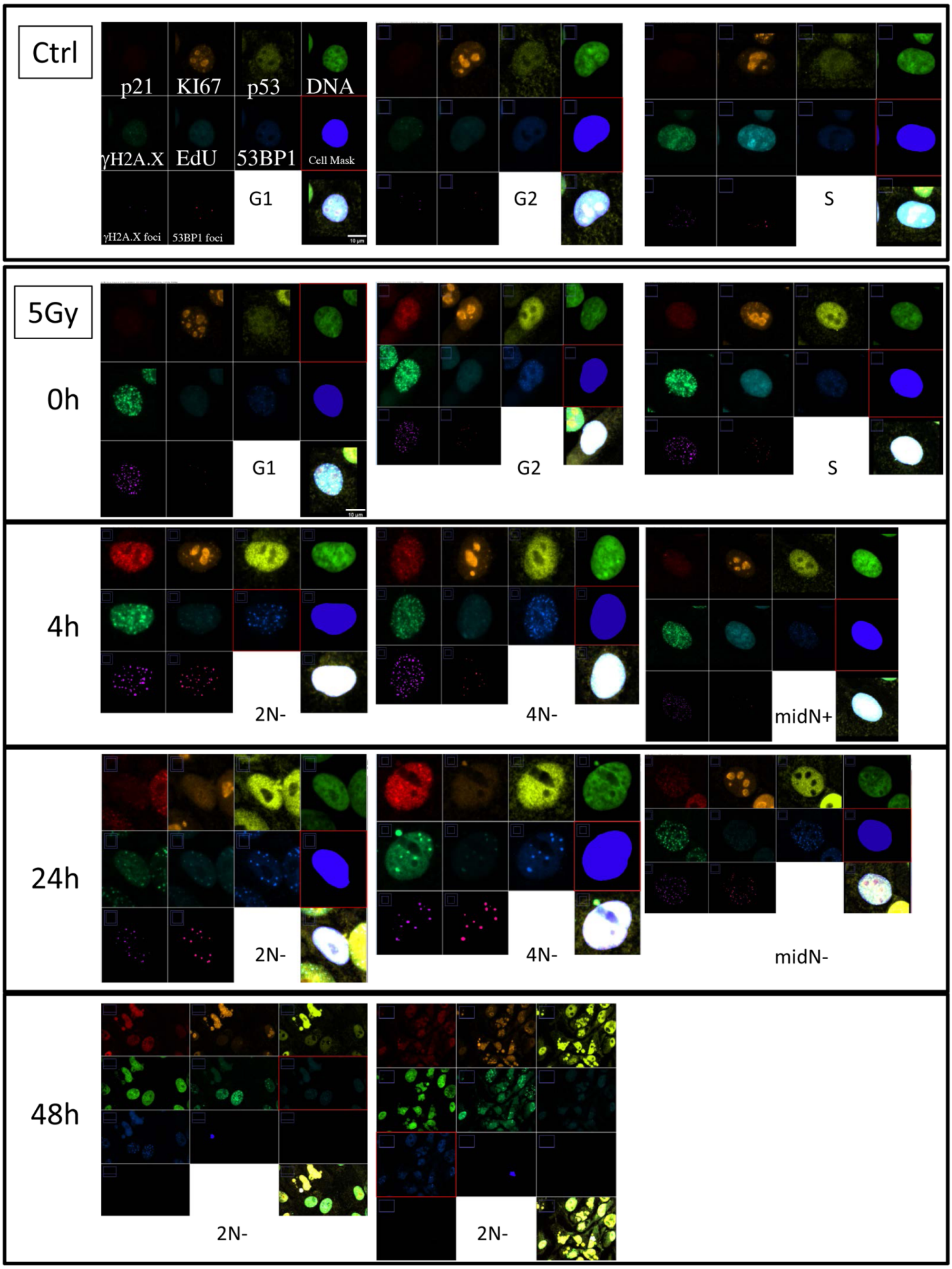

Supplementary Figure S1. Representative Images produced by the image cytometry analysis at the indicated time-points.
