# Supplementary material for: From Double-Strand Break Recognition to Cell-Cycle Checkpoint Activation: High Content and Resolution Image Cytometry Unmasks 53BP1 Multiple Roles in DNA Damage Response and p53 Action
Source: Int J Mol Sci. 2022 Sep 5;23(17):10193. doi: 10.3390/ijms231710193 (PMC9456172; doi:10.3390/ijms231710193)
Supplement: Supplementary file 1 [file ijms-23-10193-s001.zip › SupplementaryFigureS2.pdf]

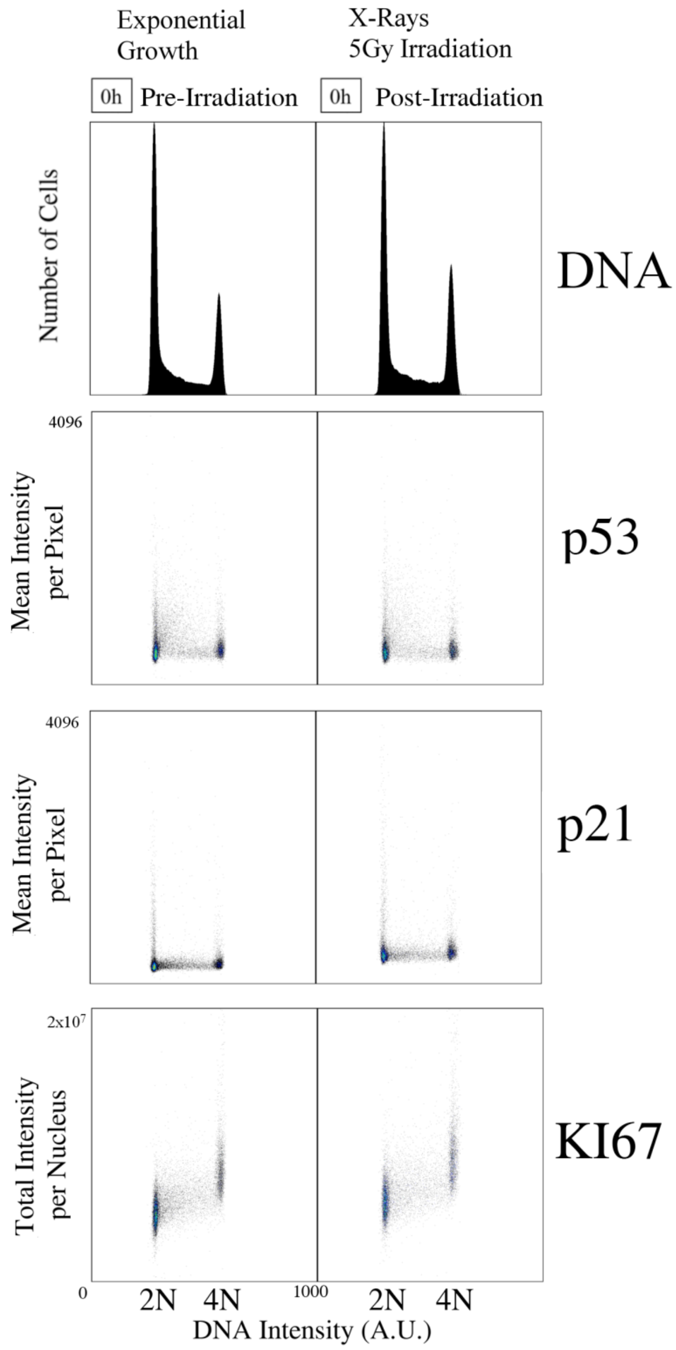

Supplementary Figure S2. DNA and protein content distribution during exponential cell growth and immediately after X-ray irradiation. The image-cytometry analysis (n>5000) shows the DNA and protein content immediately before and after (<30 minutes) X-ray irradiation. Histograms report the DNA content distribution of the entire cell population. Dot plots report the expression profile of p53, p21, and KI67 in relation to the DNA content before and immediately after the irradiation demonstrating that no changes occurred due to the short recovery time interval. Reported data refer to a representative experiment.
