# Supplementary material for: From Double-Strand Break Recognition to Cell-Cycle Checkpoint Activation: High Content and Resolution Image Cytometry Unmasks 53BP1 Multiple Roles in DNA Damage Response and p53 Action
Source: Int J Mol Sci. 2022 Sep 5;23(17):10193. doi: 10.3390/ijms231710193 (PMC9456172; doi:10.3390/ijms231710193)
Supplement: Supplementary file 1 [file ijms-23-10193-s001.zip › SupplementaryFigureS3.pdf]

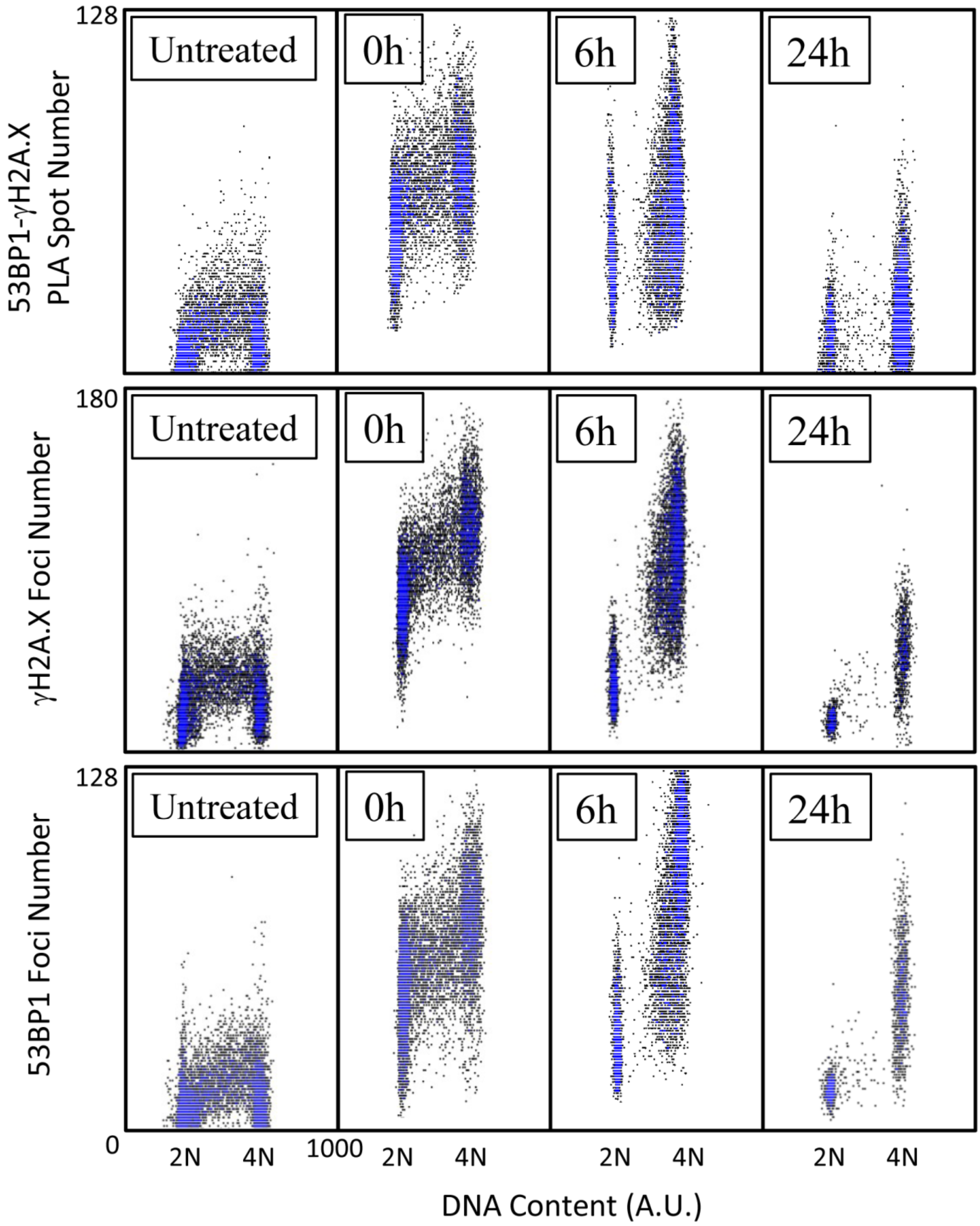

Supplementary Figure S3. 53BP1-pH2A.X PLA Analysis and DDR marker cell-cycle distribution. Cells stained for the detection of 53BP1-γH2A.X PLA spots were subjected to the described image-cytometry analysis. The distribution of the number of detected PLA spots across the cell-cycle perfectly overlap the behavior measured for the DDR foci provided by the segmentation of gH2A.X and 53BP1 spots.
