# Supplementary material for: From Double-Strand Break Recognition to Cell-Cycle Checkpoint Activation: High Content and Resolution Image Cytometry Unmasks 53BP1 Multiple Roles in DNA Damage Response and p53 Action
Source: Int J Mol Sci. 2022 Sep 5;23(17):10193. doi: 10.3390/ijms231710193 (PMC9456172; doi:10.3390/ijms231710193)
Supplement: Supplementary file 1 [file ijms-23-10193-s001.zip › SupplementaryFigureS4.pdf]

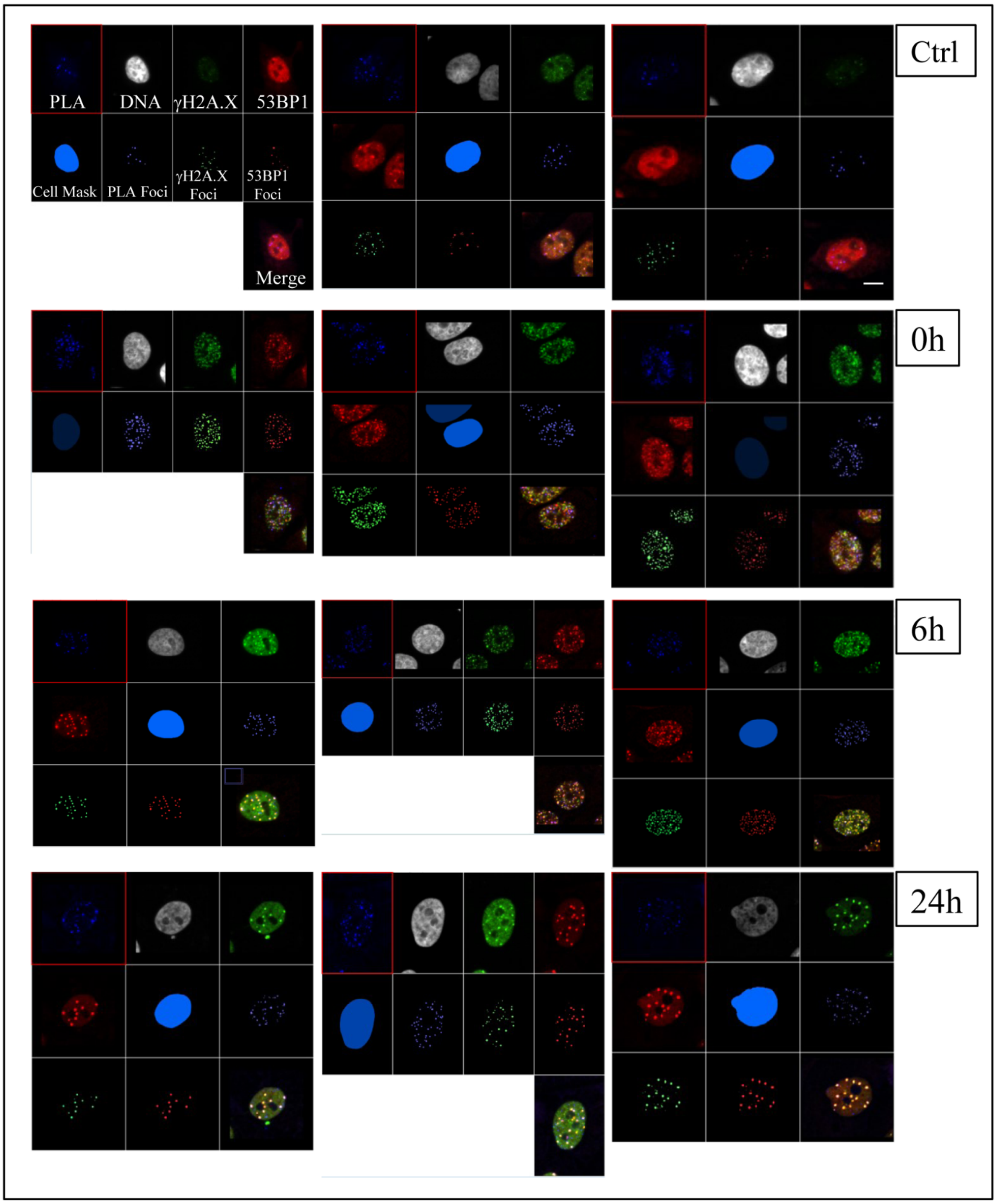

Supplementary Figure S4. Representative Images of the 53BP1-γH2A.X PLA Analysis. The reported images show the perfect colocalization of PLA and DDR foci at all the analyzed timepoints.
