# Supplementary material for: From Double-Strand Break Recognition to Cell-Cycle Checkpoint Activation: High Content and Resolution Image Cytometry Unmasks 53BP1 Multiple Roles in DNA Damage Response and p53 Action
Source: Int J Mol Sci. 2022 Sep 5;23(17):10193. doi: 10.3390/ijms231710193 (PMC9456172; doi:10.3390/ijms231710193)
Supplement: Supplementary file 1 [file ijms-23-10193-s001.zip › SupplementaryFigureS5.pdf]

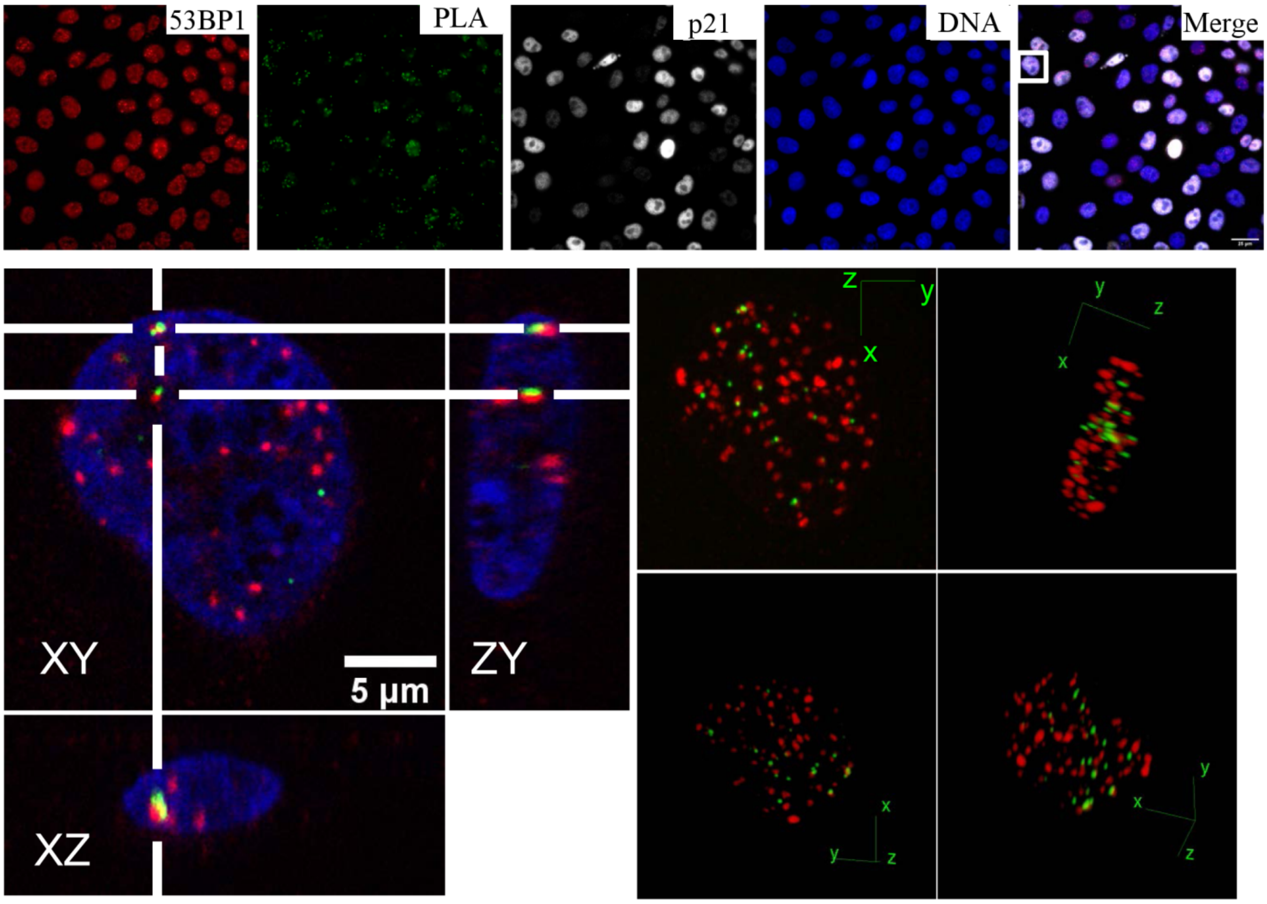

Supplementary Figure S5. Three-dimensional high-resolution confocal microscopy of 53BP1-p21 putative complex. Cells stained for the detection of 53BP1-p21 PLA spots were selected according to the described image-cytometry analysis and relocated (an exemplificative cell of interest is reported (white square) in the widefield images in the upper row; scale bar 25 microns) to perform the high resolution 3D analysis in confocal imaging. Pictures show conventional and lateral views of a selected slice (left) and 3D maximum intensity projections from different angles (right) at the indicated time-points.
